# The role of sex and gender in the changing levels of anxiety and depression during the COVID-19 pandemic: A cross-sectional study

**DOI:** 10.1177/17455065211062964

**Published:** 2021-11-30

**Authors:** Hoda Seens, Shirin Modarresi, James Fraser, Joy C MacDermid, David M Walton, Ruby Grewal

**Affiliations:** 1 Health and Rehabilitation Sciences, Elborn College, Western University, London, ON, Canada; 2 Windsor University School of Medicine, Cayon, St. Kitts; 3 School of Physical Therapy, Western University, London, ON, Canada; 4 Department of Computer Science, University of Guelph, Guelph, ON, Canada; 5 Roth McFarlane Hand and Upper Limb Centre, St. Joseph’s Health Care London, London, ON, Canada; 6 Schulich School of Medicine and Dentistry, Western University, London, ON, Canada

**Keywords:** anxiety, COVID-19, depression, gender, pandemic, sex

## Abstract

**Background:**

Several studies have assessed the impact of the COVID-19 pandemic on anxiety and depression, but have not focused on the role of sex and gender. This study compared changes in the levels of anxiety and depression (pre- and post-COVID) experienced by individuals of various sexes and genders.

**Methods:**

We used a cross-sectional online survey that assessed pre- and post-COVID symptoms of anxiety (Generalized Anxiety Disorder-2) and depression (Patient Health Questionnaire-9). General linear modeling (fixed model factorial analysis of variance) was used to evaluate changes in anxiety and depression between pre- and post-pandemic periods and explore differential effects of sex and gender on those changes.

**Results:**

Our study included 1847 participants from 43 countries and demonstrated a percentage increase of 57.1% and 74.2% in anxiety and depression, respectively. For the Generalized Anxiety Disorder-2 scale (maximum score 6), there was a mean increase in anxiety by sex for male, female, and other of 1.0, 1.2, and 1.4, respectively; and by gender for man, woman, and others of 0.9, 1.3, and 1.6, respectively. For the Patient Health Questionnaire-9 (maximum score 27), there was a mean increase in depressive symptoms by sex for male, female, and other of 3.6, 4.7, and 5.5 respectively; and by gender for man, woman, and others of 3.3, 4.8, and 6.5, respectively.

**Conclusion:**

During COVID-19, there was an increase in anxiety and depressive symptoms for all sexes and genders, with the greatest increases reported by those identifying as non-male and non-men.

## Introduction

The novel coronavirus, dubbed “COVID-19” by the World Health Organization (WHO)^1^ on 11 February 2020, was declared a pandemic on 11 March 2020.^2^ The research community in basic, clinical, and social sciences have all rushed to study the rapid physical and social perturbations. It has been demonstrated that the pandemic has led to negative psychological and social effects and that some groups are more susceptible to the negative impacts than others. For example, Pfefferbaum and North^3^ found that those who contract the virus, those with pre-existing medical or psychiatric issues, and healthcare providers were at higher risk of experiencing negative mental health as a result of the pandemic.

Sex and gender are important determinants of health. As such, both sex and gender must be considered given the biologic and social aspects of the pandemic. While the following studies have examined either sex or gender in mental health changes during the pandemic, they have not adequately distinguished between sex as a biological factor and gender as a socially constructed factor in their analysis. Wang et al.^4^ stated that “female gender” was significantly associated with greater impact on stress, anxiety, and depression levels. Mazza et al.^5^ also identified “female gender” to be associated with higher levels of stress, anxiety, and depression. Qiu et al.^6^ found that female respondents experienced higher levels of psychological distress. In a Canadian report by the Centre for Addiction and Mental Health,^7^ researchers identified that women were among a group who were more vulnerable to the symptoms of anxiety and depression during the pandemic. In a study of adults in the United States, the authors asserted that when “gender” is “female,” individuals were more susceptible to stressors during the pandemic.^8^

While these studies reinforce the need to consider sex and gender in mental health and wellness research, they have three areas of weakness. First, these studies have conflated the concept of sex and gender, often using the terms interchangeably without identifying which they have actually examined. This likely means that these separate constructs were not correctly defined or measured. Second, the studies have assumed a dichotomous classification of sex and gender while ignoring those individuals who do not identify with a binary label or who have transitioned from male to female or female to male. Finally, these studies have focused on mental health status (as measured through stress, anxiety, and depression) following the pandemic (one time point). This type of analysis merely correlates sex/gender to stressors without examining the function of the pandemic in the changing levels of stressors for those of different sexes and genders. Overall, studies have overlooked some individuals based on their gender identity and have inadequately defined sex and gender, which undermines our confidence in the findings.

Science and medicine have a long history of inadequately addressing the health needs of women and an even poorer record when it comes to genders that exist outside the classic boundaries of “men and women.” For centuries studies were conducted on men and assumed that the results could be applied with uniformity to women. As such, women have received substandard care.^9^ Increasing evidence suggests that there is a significant difference between men and women in the incidence of many diseases^10^ and response to treatment.^11^ In the arena of mental health, women have also been excluded from research because it was believed (falsely) that they were mentally inferior to men.^12^

Sex and gender are not synonymous. While sex typically refers to biological differences (i.e. genetic composition, reproductive organs, and hormones), gender is a social construct that includes the influence of environment and culture. Gender identity refers to a person’s sense of gender and may or may not correspond to the prevailing sociocultural norms attributed to one’s sex as assigned at birth.^13^ It has been suggested that socially constructed differences in roles and responsibilities interact with biological differences to create differences in the nature of mental health problems for men and women.^14^ It has also been found that women suffer more than men from internalizing disorders, which can manifest in anxiety and depression.^15^

When discussing sex and gender in mental health, we must be cognizant to include more than those who identify as binary and cisgender (whose gender identity corresponds to their sex assigned at birth).^13^ There is limited research on anxiety and depression among individuals who are not cisgender and whose gender identification could be classified as transgender. However, according to Dickey,^16^ those who do not fall into the traditional “cisgender” categories suffered from higher levels of anxiety and depression prior to the pandemic.

Knowledge of sex and gender differences in the experience of the pandemic is essential for physicians who will continue to treat patients with the mental health consequences of the pandemic. Sex and gender are important and highly personal aspects of patients’ identity and physicians must be aware of how these are related to symptoms of anxiety and depression resulting from the pandemic. Therefore, it is imperative that pandemic-related mental health research includes those who do not identify in traditionally dichotomous sex and gender classifications.

In this study, we explored differences between participants’ current ratings of anxiety and depression (which we refer to as post-COVID) and their recall of those same constructs prior to the COVID-19 pandemic (pre-COVID). We also aimed to identify how the levels of anxiety and depression have changed among individuals of various sexes and genders during the pandemic.

## Methods

### Study design

We designed the study as a cross-sectional survey to be conducted online (see supplemental material). In sections of the questionnaire where we aimed to examine changes during the pandemic, we asked respondents to answer the questions twice—first for their pre-COVID state and then for their state following the COVID-19 pandemic. We drafted the survey consisting of demographic, mental health, and homelife sections that combined standard and previously validated scales with survey-specific questions, as will be discussed below.

We designed the survey to be administered in English on the Qualtrics^17^ platform version June-July-August, 2020. The study was approved by Western University’s Health Sciences Research Ethics Board (project identification number: 115790) on 25 June 2020. For reporting transparency, we are adhering to the reporting guidelines recommended by Eysenbach^18^ in *The Checklist for Reporting Results of Internet E-Surveys (CHERRIES).* After transferring the survey into its web-based platform, its functionality was pre-tested by graduate students with clinical and methodologic expertise.

The survey was administered with the same questions, in the same order, to all participants. There were some questions with adaptive parts that were conditionally displayed depending on a response to a previous item. Other than question 1, providing informed consent to the letter of information, respondents could leave answers blank if they did not wish to answer. Respondents were able to go back in the survey to change their answers to previous questions. The Qualtrics platform uses Internet protocol addresses as a means to prevent duplicate entries from respondents. Our survey did not have a time limit for completion and respondents were given up to a 2-week period to return, complete, and submit their surveys. Respondents were also given the opportunity to enter a draw to win one of three Amazon gift cards (35 USD each).

### Participants

We used various online platforms to recruit participants between 26 June 2020 and 31 August 2020. We used social media sites (Facebook, Instagram, and Twitter), Whatsapp groups and email listservs, and community websites (Kijiji and Craigslist). Our inclusion criteria were participants who: (a) were at least 18 years of age, (b) could read and respond in English, and (c) could provide informed consent. Respondents remained anonymous throughout the study.

### Sample size

Sample size requirements were driven by our plan to conduct analysis of variance. We anticipated three groups in each of the two subsets (sex and gender), composed of the following groups: male, female, other sex, man, woman, and other gender. In order for participant groups to have sufficient power (0.8) and based on a moderate effect size (0.5) and traditionally acceptable significance level in medical research (0.05), each of the 3 groups in sex required 14 participants (total of 42 in sex) and each group in gender required 14 participants (total of 42 in gender) for a total of 84 participants.

We anticipated that not all participants would respond to all of the survey questions. Since we would not have contact information for participants, we needed to recruit a sufficient number of participants to account for approximately 50% of participants completing all necessary fields for analysis. With this more conservative estimate, we required 168 participants.

After we collected 120 questionnaires, we found the following breakdown by gender: 29 men (24.1%), 89 women (74.2%), and 2 other genders (specifically: non-binary) (1.7%). In the case of men and women, the respondents’ sex corresponded to male and female, respectively. We re-assessed our recruitment strategy at this point in the survey administration. Our goal was to obtain a sample that was diverse in sex and gender identification. Therefore, on 7 July 2020, we extended our online recruitment strategy to sex- and gender-diverse individuals through specific Facebook groups. Assuming a continuous of the same rate of respondents in other genders, in order to increase the number of respondents from 2 to 14, we would need to increase the sample size to 840. In order to obtain individuals in other sex, we would need to expand our sample even further. Our final sample size approval from Western University’s Health Sciences Research Ethics Board was 1847 participants.

### Measures

The survey consisted of the following sections: (a) consent; (b) location and job description; (c) marital status and household numbers; (d) age, sex, and gender; (e) Home and Family Work Roles Questionnaire; (f) substance use; (g) anxiety scale (Generalized Anxiety Disorder 2 (GAD-2)); (h) depression scale (Patient Health Questionnaire-9 (PHQ-9)); (i) personal COVID-19 experience; (j) physical and mental health diagnoses (Self-administered Comorbidity Questionnaire (SCQ)); and (k) additional comments and follow-up.

Some question sets were designed for this study and pilot tested. Namely, we designed sections (a) through (d), (f), (i), and (k) for this study. The Home and Family Work Roles Questionnaire (e) has been validated in an unpublished manuscript. The original version of the questionnaire contains 18 items and the version administered during the pandemic consisted of 19 items. The questionnaire examines the distribution of household responsibilities within the home by asking respondents to estimate how much of the work listed in an item they typically complete for their home or family. The GAD-2 (g) and PHQ-9 (h) scales will be discussed in detail below. SCQ (j) asks respondents to indicate if they experience a health condition from a list of common health conditions or, if not listed, to write in their health condition. If respondents pick a health condition, they would be asked if they receive treatment for it, and then if it limits their activities. This scale is widely used and has been previously validated.^19^

For this study, we analyzed the responses in GAD-2 and PHQ-9 by sex and gender. The components of GAD-2 and PHQ-9 and how we administered them will be discussed below. For the question on sex, respondents were asked “What is your sex?” The answer options were “male,” “female,” and “other (specify if you wish).” For the question on gender, respondents were asked “With which gender do you identify?” The answer options were “man,” “woman,” “non-binary,” “agender,” and “other (specify if you wish).”

#### Anxiety

We asked respondents to complete the GAD-2 scale, which contains two items. Based on this questionnaire, participants’ responses of “not at all,” “several days,” “more than half the days,” and “nearly every day” are rated on a 4-point Likert-type scale (0–3). The item scores were then added together to arrive at a total score (ranges between 0 and 6). GAD-2 is a brief patient-reported tool for screening GAD.^20^ A score of 3 or more is indicative of a clinically relevant anxiety disorder with sensitivity and specificity of 86% and 83%, respectively.^20^ In addition to screening for GAD, GAD-2 has specificity (>80%) for panic disorder, social anxiety disorder, posttraumatic stress disorder, and other anxiety disorders.^20^

We asked respondents to complete two copies of GAD-2. Respondents were instructed to fill the first copy to represent an average 2-week period before the start of the COVID-19 pandemic and the second copy to represent an average 2-week period after the start of the pandemic. We used 11 March 2020 to delineate the start of the pandemic.

#### Depression

We asked respondents to complete the PHQ-9, which contains nine items. The PHQ-9 is a screening tool for major depressive disorder (MDD) and is also used for diagnosing MDD and measuring its severity.^20^ Participants respond “not at all,” “several days,” “more than half the days,” and “nearly every day” to the items (similar to GAD-2), and the questionnaire is scored 0 to 27.^21^

Total scores in PHQ-9 may be used to indicate the severity of depression. A score of 5, 10, 15, and 20 are cut-off points for mild, moderate, moderately severe, and severe depression, respectively. When there is mild severity (scores between 5 and 9), “watchful waiting” is the proposed action.^21^ For a score of 10 or more (indicative of moderate MDD), both the sensitivity and specificity of the PHQ-9 are 88%.^22^ The PHQ-9 is also sensitive to change and able to detect when an individual’s level of MDD changes.^21^ Similar to GAD-2, we asked participants to fill two copies of PHQ-9—one for their state prior to the pandemic and the second for their state following the start of the pandemic.

### Statistical analysis

We compared participants’ level of anxiety and depression before and after the start of the COVID-19 pandemic using paired samples t-test. To evaluate the change in anxiety between different sexes and genders, we conducted mixed-model factorial analysis of variance. In this model, mean GAD-2 scores before and after (repeated measure—dependent variable) are the within-subject factors, and sex (or gender) is the between-subject factor (independent variable). If we observed a significant difference, we performed a post hoc Tukey test to assess between-group differences. We completed the same procedure for PHQ-9 scores.

We conducted all analyses using the Statistical Package for the Social Sciences (SPSS) version 26.0 program.^23^ A *p*-value of 0.05 or less was assumed to indicate statistical significance.

## Results

### Sample characteristics

The study included a total of 1847 consenting participants and a completion rate of 75.6%. We included participants with complete data for pre- and post-COVID-19 GAD-2 scales (n = 1379) and PHQ-9 scales (n = 1287) in the analysis for this article. Participants represented 43 countries of habitation, as displayed in Table 1. The age range of the sample was 18–79 years with a mean of 30.3 (± 13.3) years for GAD-2 participants and 30.4 (± 13.5) years for PHQ-9 participants, respectively.

**Table 1.** Location of participants.

Location (country/continent^a^)	Anxiety (GAD-2), *n* (%)	Depression (PHQ-9), *n* (%)
Canada	1023	951
USA	264	248
Europe	35	33
Asia	35	33
Americas	11	11
Oceania	7	7
Africa	4	4
Total	1379	1287

GAD: Generalized Anxiety Disorder; PHQ: Patient Health Questionnaire.

a Europe (Croatia, France, Germany, Ireland, Italy, Netherlands, Romania, Slovakia, Spain, Sweden, Switzerland, United Kingdom), Asia (China, India, Iran, Israel, Japan, Kazakhstan, Malaysia, Oman, Pakistan, Philippines, Qatar, Saudi Arabia, Singapore, United Arab Emirates), Americas (Antigua and Barbuda, Argentina, Bahamas, Honduras, Jamaica, Peru, Saint Kitts and Nevis), Oceania (Australia, New Zealand), and Africa (Ethiopia, Namibia, Nigeria, South Africa).

### Anxiety scale

The mean GAD-2 score was 2.1 (95% confidence interval (CI), 2.0–2.2) prior to 11 March 2020 and increased to 3.3 (95% CI, 3.2–3.4) after that date, as displayed in Table 2. This represents a mean difference of 1.2 (out of a possible 6 points) and a percentage increase of 57.1%. There was a significant difference between anxiety levels before and after the start of COVID-19 (*p* < 0.001).

**Table 2.** Symptoms of anxiety and depression.

Anxiety (GAD-2)					
N	Before (95% CI) (out of 6)	After (95% CI) (out of 6)	Mean difference	Percentage change	p-value
1379	2.1 (2.0–2.2)	3.3 (3.2–3.4)	1.2	57.1%	<0.001
Depression (PHQ-9)					
N	Before (95% CI) (out of 27)	After (95% CI) (out of 27)	Mean difference	Percentage change	p-value
1287	6.2 (6.0–6.6)	10.8 (10.4–11.1)	4.6	74.2%	<0.001

GAD: Generalized Anxiety Disorder; CI: confidence interval; PHQ: Patient Health Questionnaire.

Mean and standard deviation values for anxiety and depression scores before and after the start of the COVID-19 pandemic for all the included participants (i.e. all participants with full data for GAD-2 and PHQ-9 scales). Numbers in parentheses are 95% CIs.

**Figure 1.**
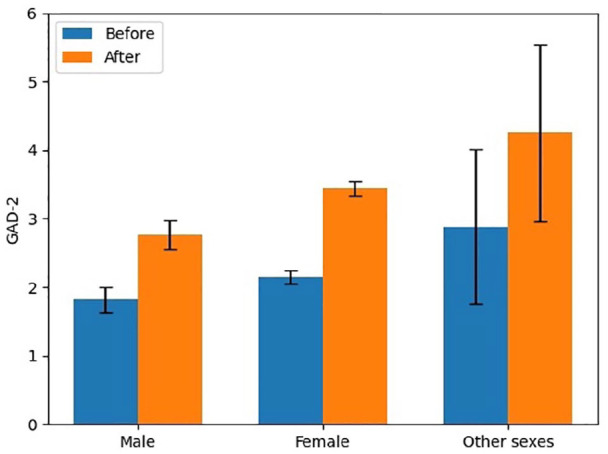
Symptoms of anxiety by sex pre- and post-COVID-19.

In the categories of sex, the frequency of respondents who reported they were male, female, and other were 21.0%, 78.4%, and 0.6%, respectively, as summarized in Table 3. The increase in anxiety scores by sex for males, females, and others was 1.0, 1.2, and 1.4 (out of a possible score of 6). In pre- and post-pandemic GAD-2 scores, males were found to have the lowest levels (from 1.8 (95% CI: 1.6–2.0) to 2.8 (95% CI: 2.6–3.0)), followed by females (from 2.2 (95% CI: 2.1–2.3) to 3.4 (95% CI: 3.3–3.6)), and the highest levels were found among those who identified as other sex (2.9 (95% CI: 1.8–4.0) to 4.3 (95% CI: 3.0–5.5)). This represented a significant sex difference in the change in GAD-2 scores (*p* < 0.001) with post hoc analysis indicating that the change in anxiety score for females (1.2) (*p* < 0.001) and those of individuals who identified as other sex (1.4) (*p* = 0.05) was significantly greater than that reported by males (1.0). There was no significant difference between change in anxiety scores of females (1.2) (*p* = 0.31) and those in the other sex category (1.4). Figure 1 illustrates a bar chart for GAD-2 scores of each sex before and after the beginning of the COVID-19 pandemic.

**Table 3.** Symptoms of anxiety and depression by sex and gender.

Anxiety (GAD-2)						
n (Frequency)	Before (95% CI) (out of 6)	After (95% CI) (out of 6)	Mean difference	Percentage change	p-value (over time)	p-value (between groups)
Male: 289 (21.0%)	1.8 (1.6–2.0)	2.8 (2.6–3.0)	1.0	55.6%	<0.001	<0.001^a^
Female: 1076 (78.4%)	2.2 (2.1–2.3)	3.4 (3.3–3.6)	1.2	54.5%	<0.001	0.31^b^
Other: 8 (0.6%)	2.9 (1.8–4.0)	4.3 (3.0–5.5)	1.4	48.3%	0.05	0.05^c^
Man: 283 (20.6%)	1.8 (1.6–2.0)	2.7 (2.5–3.0)	0.9	50.0%	<0.001	<0.001^d^
Woman: 1058 (77.1%)	2.1 (2.0–2.2)	3.4 (3.3–3.5)	1.3	61.9%	<0.001	0.02^e^
Other genders: 31 (2.6%)	2.7 (2.2–3.3)	4.3 (3.6–4.9)	1.6	59.3%	<0.001	<0.001^f^
Depression (PHQ-9)						
n (Frequency)	Before (95% CI) (out of 27)	After (95% CI) (out of 27)	Mean difference	Percentage change	p-value (over time)	p-value (between groups)
Male: 273 (21.3%)	6.0 (5.4–6.7)	9.6 (8.8–10.3)	3.6	60.0%	<0.001	0.04^a^
Female: 1000 (78.1%)	6.3 (5.9–6.6)	11.0 (10.6–11.5)	4.7	74.6%	<0.001	0.03^b^
Other: 8 (0.6%)	10.8 (7.0–14.5)	16.3 (11.6–20.9)	5.5	50.9%	0.05	0.007^c^
Man: 268 (20.9%)	6.1 (5.4–6.7)	9.4 (8.6–10.2)	3.3	54.1%	<0.001	0.05^d^
Woman: 984 (76.9%)	6.2 (5.9–6.5)	11.0 (10.5–11.4)	4.8	77.4%	<0.001	<0.001^e^
Other genders: 28 (2.2%)	9.8 (7.8–11.7)	16.3 (13.9–18.8)	6.5	66.3%	<0.001	<0.001^f^

GAD: Generalized Anxiety Disorder; CI: confidence interval; PHQ: Patient Health Questionnaire.

Mean and standard deviation values for anxiety and depression scores before and after the start of the COVID-19 pandemic by sex and gender.

Numbers in parentheses are 95% CIs.

a Between male and female.

b Between female and other.

c Between other and male.

d Between man and woman.

e Between woman and other genders.

f Between other gender and man.

**Figure 2.**
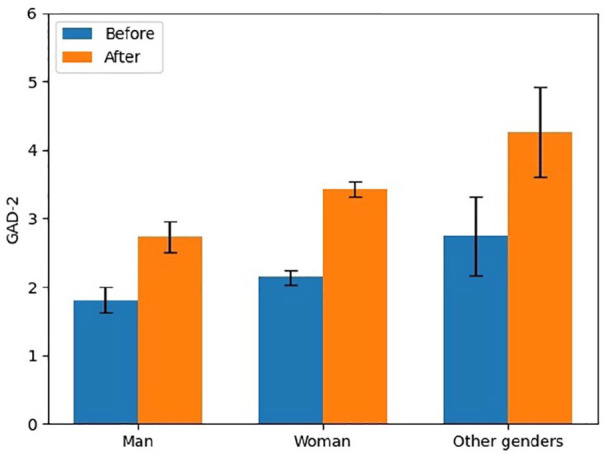
Symptoms of anxiety by gender pre- and post-COVID-19.

For gender, the frequency of respondents who reported identifying as men, women, and other genders (including non-binary, agender, other) was 20.6%, 77.1%, and 2.6%, respectively. The increase in GAD-2 scores by gender for men, women, and other genders was 0.9, 1.3, and 1.5 (out of a possible 6 points), respectively. In pre- and post-pandemic GAD-2 scores, men were found to have the lowest levels (from 1.8 (95% CI: 1.6–2.0) to 2.7 (95% CI: 2.5–3.0)), followed by women (from 2.1 (95% CI: 2.0–2.2) to 3.4 (95% CI: 3.3–3.5)), and the highest levels were found among those who identified as other genders (2.7 (95% CI: 2.2–3.3) to 4.3 (95% CI: 3.6–4.9)). There was a significant gender difference in the change in GAD-2 scores (*p* < 0.001). Post hoc analysis revealed that the change in anxiety score for women (1.3) (*p* < 0.001) and those of people who identified in other genders (1.6) (*p* < 0.001) was significantly larger than that reported by men (0.9). Other genders’ change in GAD-2 scores (1.6) (*p* = 0.02) was significantly greater than that reported by women (1.3). Figure 2 illustrates a bar chart for GAD-2 scores of each gender before and after the beginning of the COVID-19 pandemic.

### Depression scale

The mean PHQ-9 score was 6.2 (95% CI: 6.0–6.6) prior to 11 March 2020 and increased to 10.8 (95% CI: 10.4–11.1) after that date, as displayed in Table 2. This represents a mean difference of 4.6 (out of a possible 27 points) and a percentage increase of 74.2%. There was a significant difference between depression levels before and after the start of COVID-19 (*p* < 0.001).

**Figure 3.**
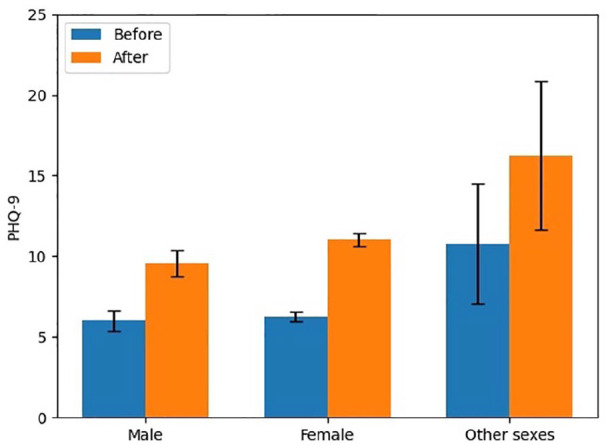
Symptoms of depression by sex pre- and post-COVID-19.

In the categories of sex, the frequency of respondents who reported they were male, female, and other were 21.3%, 78.1%, and 0.6%, respectively, as summarized in Table 3. The increase in depression scores by sex for males, females, and others was 3.6, 4.7, and 5.5 (out of a possible score of 27). In pre- and post-pandemic PHQ-9 scores, males were found to have the lowest levels (from 6.0 (95% CI: 5.4–6.7) to 9.6 (95% CI: 8.8–10.3)), followed by females (from 6.3 (95% CI: 5.9–6.6) to 11.0 (95% CI: 10.6–11.5)), and the highest levels were found among those who identified as other sex (10.8 (95% CI: 7.0–14.5) to 16.3 (95% CI: 11.6–20.9)). This represents a significant sex difference in the change in PHQ-9 scores (*p* = 0.001) with post hoc analysis indicating that change in levels of depression for females (4.7) (*p* = 0.04) and those of people that identified as other sex (5.5) (*p* = 0.007) was significantly greater than that reported by males (3.6). There was also a significant difference between depression scores of other sex (5.5) (*p* = 0.03) and females (4.7). Figure 3 illustrates a bar chart for PHQ-9 scores of each sex before and after the beginning of the COVID-19 pandemic.

**Figure 4.**
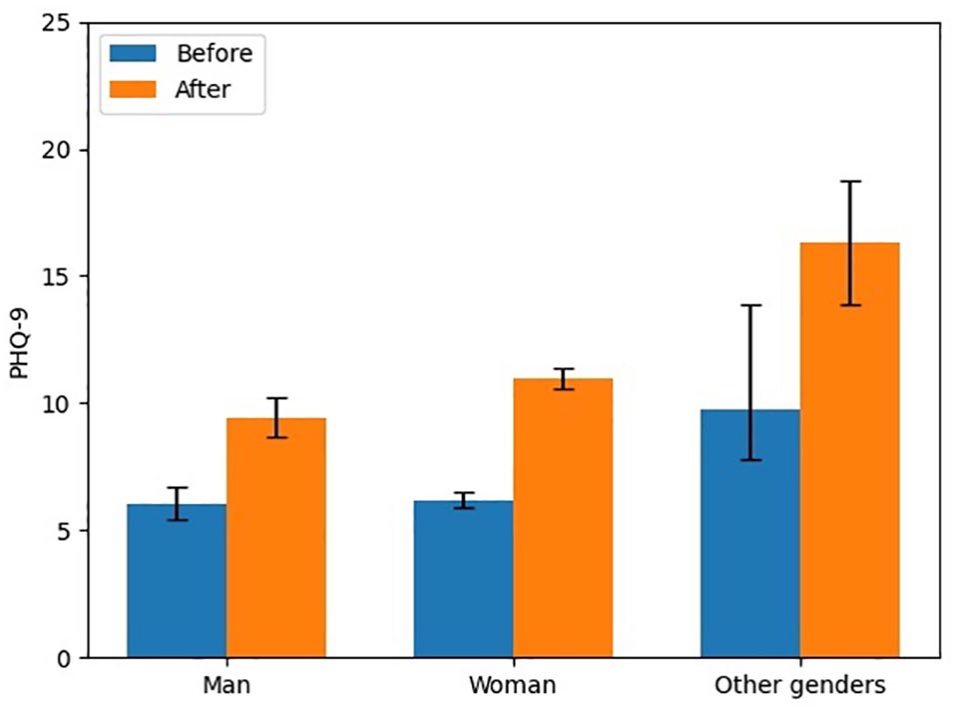
Symptoms of depression by gender pre- and post-COVID-19.

For gender, the frequency of respondents who reported identifying as men, women, and other genders was 20.9%, 76.9%, and 2.2%, respectively. The increase in PHQ-9 scores by gender for men, women, and other genders was 3.3, 4.8, and 6.5 (out of a possible 27 points), respectively. In pre- and post-pandemic PHQ-9 scores, men were found to have the lowest levels (from 6.1 (95% CI: 5.4–6.7) to 9.4 (95% CI: 8.6–10.2)), followed by women (from 6.2 (95% CI: 5.9–6.5) to 11.0 (95% CI: 10.5–11.4)), and the highest levels were found among those who identified as other genders (9.8 (95% CI: 7.8–11.7) to 16.3 (95% CI: 13.9–18.8)). There was a significant gender difference in change in PHQ-9 scores (*p* < 0.001). Post hoc analysis revealed that women’s increase in depression (4.8) (*p* = 0.05) and those of other genders (6.5) (*p* < 0.001) was significantly more than that reported by men (3.3). Other genders’ change in PHQ-9 scores (6.5) (*p* < 0.001) was significantly greater than that reported by women (4.8). Figure 4 illustrates a bar chart for PHQ-9 scores of each gender before and after the beginning of the COVID-19 pandemic.

## Discussion

### Implications

Our results indicate that the levels of anxiety have increased following the pandemic by 57.1% for the study sample when comparing pre- and post-COVID scores. The mean GAD-2 score increased from 2.1 to 3.3 for respondents. Based on a cut-point score of 3 for GAD-2,^20^ our results indicate the clinical significance of this finding since it suggests a mean increase from an absence of anxiety to the presence of anxiety, in pre-post pandemic scores. Our results also indicate an increased level of depression of 74.2% in the study sample. The mean PHQ-9 score increased from 6.2 to 10.8 for respondents. Based on a cut-point score of 10 (requiring treatment for depression) for PHQ-9,^22^ our results also indicate the clinical significance of this finding since it suggests a mean increase from no treatment required to treatment required, in pre-post pandemic scores.

Our findings that anxiety and depression have increased during the pandemic are consistent with many other population-based studies.^4–6,8,24,25^ What this study adds is new data on sex and gender differences in changes in mental health. After the start of the pandemic, all sexes except males and all genders except men met the cut-off for having an anxiety disorder. This is both due to higher pre-COVID levels among non-male and non-man respondents and a greater mean difference in scores. A similar trend in the increased levels of depression exists among the sexes and genders with respect to depression following the pandemic, with non-male and non-man categories meeting the cut-off for moderate MDD (signaling a clinical shift).

Although the literature often conflates sex and gender,^13^ we know that females experience anxiety and depression at a higher prevalence than males.^26^ We also know that it can be difficult to separate the biological sex factors from the social gender factors that, individually or in combination, affect mental health. However, since the differences in anxiety and depressive scores were generally larger for gender subgroups than sex subgroups, this indicates that social factors are important. This does not preclude sex differences in susceptibility to anxiety and depression that might arise due to hormonal, brain structure and function, or other factors.

We hypothesized that females and women might be experiencing greater increases in anxiety and depressive symptoms during the pandemic because of the feminine tendency in mental health toward internalizing disorders.^15^ If this trend at internalization versus externalization remains consistent during the pandemic, it may be expected that males and men will have greater use of substances (such as alcohol and recreational drugs) following the pandemic, which we will examine in future studies. Another contributing factor to our observed trends may be that, with school closures and families being at home, the historically gendered roles of childcare and home-making have fallen more heavily on those who do not self-report as being males or men. Finally, women may be more dependent on social support and opportunities to talk to other adults outside their immediate household. Research indicates that women are more likely than men to care for those outside of their own homes.^27^ As such, women may be more accustomed to interactions outside of the immediate household for maintenance of mental health, and social isolation may more directly impact this coping mechanism.

Our findings indicate that those who identified as other in sex and gender had the greatest mean difference in GAD-2 and PHQ-9 scores following the pandemic even though they also had the highest pre-COVID scores. This may be explained, in part, by the Minority Stress Model^28^ where individuals who have minority status (including in sex and gender) may experience stigma, discrimination, and oppression compared to their majority counterparts. This, in turn, can increase levels of distress and contribute to mental health disorders. In addition, during the pandemic, many individuals may have had a sense of isolation as they spend time at home and in their households. In a study of sexual and gender minority individuals, community connectedness had a moderating effect on anxiety and depression.^29^ Therefore, isolation may have been especially distressing to sex and gender marginalized individuals if it disrupted social circles or forced segregation with unsupportive families.

### Strengths and limitations

Our study has several strengths including its large sample, which includes diversity in geography, sex, gender, and age. Our survey was anonymously administered to minimize stigma or social desirability bias in responses. However, our results should also be interpreted considering some limitations. First, the study was completed on the Internet where there is a risk of selection bias, for example: exclusion of those from lower socioeconomic, older, or less educated subgroups of the population. Second, the study was designed as a cross-sectional survey with a retrospective component and participants had to report their pre-pandemic status retrospectively. This has the potential for recall bias, although we were most interested in their change scores and it is likely that participants calibrated their response to how much they felt their status had changed. Third, although we used a variety of strategies to facilitate recruitment of non-binary sexes and genders and gathered data from individuals with various gender identifications (non-binary, agender, other), we were only able to analyze the data by grouping these various genders into one “other” category because we did not achieve sufficient power for more definitive description. For sex, we were unable to achieve our predetermined power (0.8) with the sample of respondents (n = 8) who identified in the other sex category. However, we chose to present these findings in our results. Although the sample in the other sex category may be small, it is an important and often marginalized group that has been historically ignored in research. Ethically, we maintained the findings from this group as a separate and distinct category in our results. Fourth, respondent’s culture (both their country of habitation and ethnic identification) may be closely intertwined with sex and gender identification and experiences, which we did not analyze in this study.

## Conclusion

Our research has determined that individuals of varying sexes and genders are experiencing increased symptoms of anxiety and depression when comparing pre- and post-COVID status (as measured through GAD-2 and PHQ-9). It also indicates that more marginalized genders had greater increases in symptoms. Therefore, we need to be aware of these differences, identify their underlying causes, and work to help individuals achieve the best state of mental health during and after the pandemic.

## Supplemental Material

sj-docx-1-whe-10.1177_17455065211062964 – Supplemental material for The role of sex and gender in the changing levels of anxiety and depression during the COVID-19 pandemic: A cross-sectional study Click here for additional data file. Supplemental material, sj-docx-1-whe-10.1177_17455065211062964 for The role of sex and gender in the changing levels of anxiety and depression during the COVID-19 pandemic: A cross-sectional study by Hoda Seens, Shirin Modarresi, James Fraser, Joy C MacDermid, David M Walton and Ruby Grewal in Women’s Health
