# Supplementary material for: The role of sex and gender in the changing levels of anxiety and depression during the COVID-19 pandemic: A cross-sectional study
Source: Womens Health (Lond). 2021 Nov 30;17:17455065211062964. doi: 10.1177/17455065211062964 (PMC8640979; doi:10.1177/17455065211062964)
Supplement: sj-docx-1-whe-10.1177_17455065211062964 – Supplemental material for The role of sex and gender in the changing levels of anxiety and depression during the COVID-19 pandemic: A cross-sectional study [file sj-docx-1-whe-10.1177_17455065211062964.docx]

Mental Health amid COVID-19

Start of Block: Consent

Q1
**Letter of Information and Consent**
**Study Title: Mental Health amid COVID-19 (Survey)**

 **Investigators**

 **Dr. Joy MacDermid, PT PhD (Principal Investigator)**
 Department of Physical Therapy, Western University
                                                                                                             
 **Ms. Hoda Seens, PhD candidate (Co-investigator)**
 Health and Rehabilitation Sciences, Western University                                       
 Email: [hmalakou@uwo.ca](mailto:cziebart@uwo.ca)                                                                                      

                         
 **Study Purpose**
The COVID-19 pandemic has affected our lives in many ways. The purpose of this study is to understand how the pandemic has affected some aspects of our mental health.
We would like to explore if and how the pandemic has affected you in terms of family responsibilities, anxiety, depression, and the use of substance (such as caffeine and tobacco).


 **Procedures**
 This study is a questionnaire. Please read through this study information. If you have any questions, do not hesitate to contact Hoda for clarification.
 It will take approximately 20 - 30 minutes to complete the questionnaire.


 **Who can Participate**
 If you are aged 18 years or older and can read and understand English, you may choose to participate.
 We are looking for approximately 1800 individuals to respond to the survey.
  

 **Participating in the Study**
 Participating in this study is voluntary. You may refuse to participate, refuse to answer any questions, or stop answering the survey.
You do not waive any legal rights by agreeing to participate in this study.


 **Withdrawing from the Study**
You may decide to stop answering the survey at any time. If you decide to withdraw from the study, the information that was collected up to that point can still be used to answer the research question. The survey data is completely anonymous so individual answers cannot be identified and removed. If you decide to withdraw, you will not be asked to provide any more information.


 **Benefits**
There are no direct benefits to you. But your study participation will have societal benefits by helping improve knowledge about mental health during pandemics and the resources we may need to cope.


 **Risks**
Thinking about the COVID-19 pandemic and its effects may be upsetting. If during the course of the questionnaire you are upset, you may stop taking the questionnaire. Please consult with your health care provider or public health unit if you feel that you need help in coping.


 **Compensation**
There is no monetary reimbursement for participation in this study. But, as a token of our gratitude, you will have an opportunity to submit your email address for a chance to win one of three Amazon gift cards (50 CAD or 35 USD).


 **Confidentiality**
Your individual results will be held in strict confidence.
No person, other than the study team, will have access to the data.
Representatives of Western University Health Sciences Research Ethics Board may require access to study records to monitor the conduct of the research.
By Western University policy, we will retain study-related data (on a secure Western network) for 7 years, at which time it will be permanently destroyed.

All email addresses collected for the gift draw, through the separate link, will only be used for the purpose of the draw. We will delete these email addresses once gift cards have been distributed. The link to provide your email address for the draw is completely separate from this questionnaire and cannot be connected to you or your responses.


**Questions about your Rights**
If you have any questions about your rights as a research participant, you may contact The Office of Human Research Ethics at ethics@uwo.ca or 519-661-3036.


 **Questions about the Study**
If you have any questions about the study, contact Hoda Seens (co-investigator) at hmalakou@uwo.ca.


 **Consent**
 If you would like to voluntarily participate, please indicate that you are providing consent:

- Yes, I consent to participate (1)
- No, I do not consent (2)

Skip To: End of Survey If PLEASE READ THE STUDY INFORMATION BELOW AND PROVIDE YOUR CONSENT TO PARTICIPATE: Study Title = No, I do not consent

End of Block: Consent

Start of Block: Location_job

Q2 Where do you live?

Country (1)

Province/State (2)

▼ Country (1) ... Zimbabwe ~ Midlands Province (3583)

| 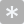 |
| --- |

Q3 Which city do you live in?

________________________________________________________________

Q4 Which statement best describes your current employment status?

- Paid employee (8)
- Self-employed (9)
- Laid off (11)
- Stay-at-home parent/caretaker (12)
- Student (13)
- Retired (14)
- Unable to work due to a disability (15)
- Other (please specify) (16) ________________________________________________

Display This Question:

If Which statement best describes your current employment status? = Paid employee

Or Which statement best describes your current employment status? = Self-employed

Q5 What is your job title?

________________________________________________________________

Display This Question:

If Which statement best describes your current employment status? = Laid off

Q6 What was your job?

________________________________________________________________

Display This Question:

If Which statement best describes your current employment status? = Student

Q7 What do you study?

________________________________________________________________

Q8 Prior to the pandemic, did you have a paid job?

- Yes, full time (more than 20 hours/week) (1)
- Yes, part time (20 or fewer hours/week) (2)
- No (3)
- Other (please specify) (4) ________________________________________________

Q9 Since the pandemic started, do you have a paid job?

- Yes, full time (more than 20 hours/week) (1)
- Yes, part time (20 or fewer hours/week) (2)
- No (3)
- Other (please specify) (4) ________________________________________________

Display This Question:

If Since the pandemic started, do you have a paid job? = Yes, full time (more than 20 hours/week)

Or Since the pandemic started, do you have a paid job? = Yes, part time (20 or fewer hours/week)

Q10 As a result of the pandemic, do you have to work from home?

- With a similar workload (1)
- With a greater workload (2)
- With a lighter workload (3)
- I still go into my work place (4)
- I lost my job (5)
- I have always worked from home (6)

End of Block: Location_job

Start of Block: Household

Q11 What is your marital status?

- Single (1)
- Common-law (2)
- Married (3)
- Divorced (4)
- Widowed (5)
- Other (please specify) (6) ________________________________________________

Display This Question:

If What is your marital status? = Common-law

Or What is your marital status? = Married

Q12 What is the gender of your partner?

- Man (1)
- Woman (2)
- Non-binary (4)
- Agender (6)
- Other (specify if you wish) (3) ________________________________________________

Q13 How many people in total currently live in your home, including yourself?

▼ Select an answer (2) ... > 12 (15)

Q14 Has the number of people living in your home changed as a result of the pandemic?

- Yes (1)
- No (2)

Display This Question:

If Has the number of people living in your home changed as a result of the pandemic? = Yes

Q15 Please pick all the ways in which the number of people in your household has changed as a result of the pandemic.

- Increased - elder(s) moved in (1)
- Increased - adult child(ren) moved in (2)
- Decreased - family member(s) with high exposure risk to COVID-19 moved out (3)
- Other - please specify (4) ________________________________________________

Q16 How many children (who are dependent on you) currently live in your home?

▼ Select an answer (1) ... > 10 (13)

End of Block: Household

Start of Block: Age_gender

Q17 What is your age?

▼ 18 (2) ... 105 (89)

Q18 What is your sex?

- Male (1)
- Female (2)
- Other (specify if you wish) (3) ________________________________________________

Q19 With which gender do you identify?

- Man (1)
- Woman (2)
- Non-binary (4)
- Agender (5)
- Other (specify if you wish) (3) ________________________________________________

Q20 With which ethnic origin (or visibility) do you identify?

Please mark all that apply.

- North American Aboriginal (1)
- Black (8)
- White (5)
- Arab (16)
- Latin, Central, and South American (9)
- South Asian (E.g. East Indian, Pakistani) (10)
- West Asian (E.g. Iranian, Afghan) (11)
- Southeast Asian (E.g. Vietnamese, Cambodian) (12)
- East Asian (E.g. Chinese, Japanese, Korean) (13)
- Pacific Islands (E.g. Fijian, Hawaiian) (14)
- Other (please specify) (15) ________________________________________________

End of Block: Age_gender

Start of Block: Family_responsibility

Q21 Think about the work you did to take care of your home and family BEFORE the COVID-19 pandemic (before March 11, 2020). Please do not count the work done by anyone else (family, friends, spouses, paid staff, etc.). Slide the scale to represent the percent of the work you did. If the question does not apply to you (example: you do not have children), then choose "not applicable."

|  | Percentage of work | Not Applicable |
| --- | --- | --- |

|  | 0 | 10 | 20 | 30 | 40 | 50 | 60 | 70 | 80 | 90 | 100 |
| --- | --- | --- | --- | --- | --- | --- | --- | --- | --- | --- | --- |

| House cleaning (floors, dishes, bathrooms, etc.) () | 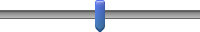 |
| --- | --- |
| Outdoor cleaning (garage, garbage, windows, etc.) () | 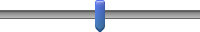 |
| Laundry () | 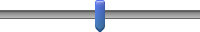 |
| Home decorating (painting, wallpapering, etc.) () | 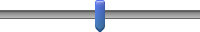 |
| Home repairs (install doors or lights, fix bathroom, etc.) () | 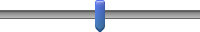 |
| Mow lawn () | 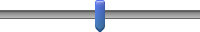 |
| Garden (plant, weed, etc.) () | 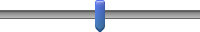 |
| Prepare meals () | 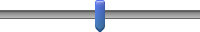 |
| Shop for groceries and supplies () | 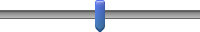 |
| Drive family to appointments and activities () | 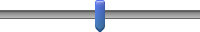 |
| Arrange family appointments and activities () | 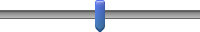 |
| Maintain vehicles (repair, change oil, clean, etc.) () | 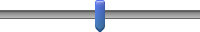 |
| Help children with homework () | 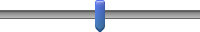 |
| Supervise children with homework () | 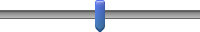 |
| Care for children in the home () | 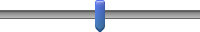 |
| Care for children when sick () | 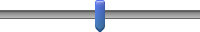 |
| Care for other family members (parent, spouse, or others) () | 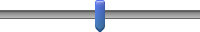 |
| Earn family income () | 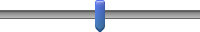 |
| Manage family finances/bills () | 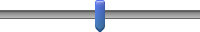 |

Q22 Similar to the previous question, think about the work you did to take care of your home and family AFTER the COVID-19 pandemic began (after March 11, 2020).

|  | Percentage of Work | Not Applicable |
| --- | --- | --- |

|  | 0 | 10 | 20 | 30 | 40 | 50 | 60 | 70 | 80 | 90 | 100 |
| --- | --- | --- | --- | --- | --- | --- | --- | --- | --- | --- | --- |

| House cleaning (floors, dishes, bathrooms, etc.) () | 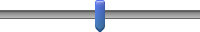 |
| --- | --- |
| Outdoor cleaning (garage, garbage, windows, etc.) () | 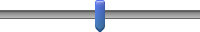 |
| Laundry () | 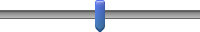 |
| Home decorating (painting, wallpapering, etc.) () | 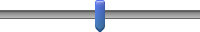 |
| Home repairs (install doors or lights, fix bathroom, etc.) () | 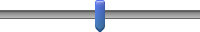 |
| Mow lawn () | 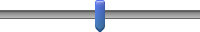 |
| Garden (plant, weed, etc.) () | 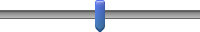 |
| Prepare meals () | 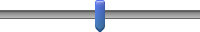 |
| Shop for groceries and supplies () | 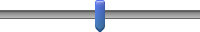 |
| Drive family to appointments and activities () | 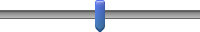 |
| Arrange family appointments and activities () | 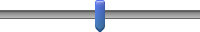 |
| Maintain vehicles (repair, change oil, clean, etc.) () | 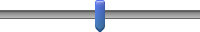 |
| Help children with homework () | 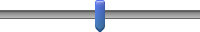 |
| Supervise children with homework () | 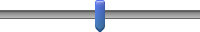 |
| Care for children in the home () | 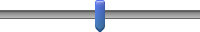 |
| Care for children when sick () | 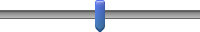 |
| Care for other family members (parent, spouse, or others) () | 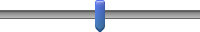 |
| Earn family income () | 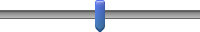 |
| Manage family finances/bills () | 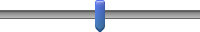 |

End of Block: Family_responsibility

Start of Block: Substance_use

Q23
In the following four questions, we would like to know how the COVID-19 pandemic has affected your daily use of substances (caffeine, tobacco, alcohol, and recreational drugs). Please be assured that your responses are completely confidential. For each question, select your average use BEFORE and AFTER the start of the pandemic (using March 11, 2020 as the pandemic date).


How many caffeinated beverages do you drink per day? Please count cups/cans/bottles of coffee, pop/soda, energy drinks, etc.

|  |  |
| --- | --- |
| Before the pandemic (1) | ▼ 0 (1) ... > 10 (14) |
| Since the pandemic (2) | ▼ 0 (1) ... > 10 (14) |

Q24 How many packs of cigarettes do you smoke per day?

|  |  |
| --- | --- |
| Before the pandemic (1) | ▼ 0 (6) ... > 2 (5) |
| Since the pandemic (4) | ▼ 0 (6) ... > 2 (5) |

Q25 How many glasses of alcohol (wine, beer, hard liquor) do you drink per day?

|  |  |
| --- | --- |
| Before the pandemic (1) | ▼ 0 (3) ... > 8 (12) |
| Since the pandemic (4) | ▼ 0 (3) ... > 8 (12) |

Q26 Do you use any recreational drugs?

|  |  |
| --- | --- |
| Before the pandemic (7) | ▼ None (1) ... Multiple drugs (7) |
| Since the pandemic (8) | ▼ None (1) ... Multiple drugs (7) |

End of Block: Substance_use

Start of Block: Anxiety

Q27
Please indicate how often you were bothered by the following problems for an average two-week period BEFORE the pandemic. Then indicate how often you are bothered by these problems for an average two-week period AFTER the start of the pandemic (using March 11, 2020 as the pandemic date).


If you are using a mobile device to complete this survey, please make sure to scroll across each row.

|  | Before the pandemic | | | | Since the pandemic | | | |
| --- | --- | --- | --- | --- | --- | --- | --- | --- |
|  | Not at all (1) | Several days (2) | More than half the days (3) | Nearly every day (4) | Not at all (1) | Several days (2) | More than half the days (3) | Nearly every day (4) |
| Feeling nervous, anxious or on edge (1) |  |  |  |  |  |  |  |  |
| Not being able to stop or control worrying (2) |  |  |  |  |  |  |  |  |
| Worrying too much about different things (3) |  |  |  |  |  |  |  |  |
| Trouble relaxing (4) |  |  |  |  |  |  |  |  |
| Being so restless that it is hard to sit still (5) |  |  |  |  |  |  |  |  |

End of Block: Anxiety

Start of Block: Depression

Q28
Please indicate how often you were bothered by the following problems for an average two-week period BEFORE the pandemic. Then indicate how often you are bothered by these problems for an average two-week period AFTER the start of the pandemic (using March 11, 2020 as the pandemic date).


If you are using a mobile device to complete this survey, please make sure to scroll across each row.

|  | Before the pandemic | | | | Since the pandemic | | | |
| --- | --- | --- | --- | --- | --- | --- | --- | --- |
|  | Not at all (1) | Several days (2) | More than half the days (3) | Nearly every day (4) | Not at all (1) | Several days (2) | More than half the days (3) | Nearly every day (4) |
| Little interest or pleasure in doing things (1) |  |  |  |  |  |  |  |  |
| Feeling down, depressed, or hopeless (8) |  |  |  |  |  |  |  |  |
| Trouble falling or staying asleep, or sleeping too much (2) |  |  |  |  |  |  |  |  |
| Feeling tired or having little energy (3) |  |  |  |  |  |  |  |  |
| Poor appetite or overeating (4) |  |  |  |  |  |  |  |  |
| Feeling bad about yourself - or that you are a failure or have let yourself or your family down (5) |  |  |  |  |  |  |  |  |
| Trouble concentrating on things, such as reading the newspaper or watching television (6) |  |  |  |  |  |  |  |  |
| Moving or speaking so slowly that other people could have noticed? Or the opposite - being so fidgety or restless that you have been moving around a lot more than usual (7) |  |  |  |  |  |  |  |  |
| Thoughts that you would be better off dead or of hurting yourself in some way (9) |  |  |  |  |  |  |  |  |

Display This Question:

If Please indicate how often you were bothered by the following problems for an average two-week per... : Before the pandemic = Several days

And Please indicate how often you were bothered by the following problems for an average two-week per... : Before the pandemic = Several days

Or Please indicate how often you were bothered by the following problems for an average two-week per... : Before the pandemic = More than half the days

Or Please indicate how often you were bothered by the following problems for an average two-week per... : Before the pandemic = Nearly every day

Or Please indicate how often you were bothered by the following problems for an average two-week per... : Since the pandemic = Several days

Or Please indicate how often you were bothered by the following problems for an average two-week per... : Since the pandemic = More than half the days

Or Please indicate how often you were bothered by the following problems for an average two-week per... : Since the pandemic = Nearly every day

Q29 If you checked off ANY problems from the previous question, how difficult have these problems made it for you to do your work, take care of things at home, or get along with others?

|  |  |
| --- | --- |
| Before the pandemic (1) | ▼ Impossible (9) ... Not at all (6) |
| Since the pandemic (4) | ▼ Impossible (9) ... Not at all (6) |

End of Block: Depression

Start of Block: Covid

Q30 Do you personally know someone who has tested positive for COVID-19?

- Yes (1)
- No (2)
- I'm not sure (3)

Display This Question:

If Do you personally know someone who has tested positive for COVID-19? = Yes

| 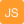 |
| --- |

Q31 How do you know this person(s) with COVID-19?

- Myself (11)
- My partner (2)
- My child(ren) (3)
- My parent(s) (4)
- Other family member(s) (please specify) (5) ________________________________________________
- Friend(s) (6)
- Acquaintance(s) (7)
- Coworker(s) (8)
- Classmate(s) (9)
- Other (please specify) (10) ________________________________________________

Display This Question:

If If How do you know this person(s) with COVID-19? q://QID125610082/SelectedChoicesCount Is Greater Than 1

And How do you know this person(s) with COVID-19? = Myself

Or How do you know this person(s) with COVID-19? != Myself

And And How do you know this person(s) with COVID-19? q://QID125610082/SelectedChoicesCount Is Greater Than 0

Carry Forward Selected Choices - Entered Text from "How do you know this person(s) with COVID-19?"

| 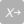 |
| --- |

Q32 Was this person(s) living in a long-term care facility?

|  | Selection | | |
| --- | --- | --- | --- |
|  | Yes (1) | No (2) | I don't know (3) |
| Myself (x11) |  |  |  |
| My partner (x2) |  |  |  |
| My child(ren) (x3) |  |  |  |
| My parent(s) (x4) |  |  |  |
| Other family member(s) (please specify) (x5) |  |  |  |
| Friend(s) (x6) |  |  |  |
| Acquaintance(s) (x7) |  |  |  |
| Coworker(s) (x8) |  |  |  |
| Classmate(s) (x9) |  |  |  |
| Other (please specify) (x10) |  |  |  |

| Page Break |  |
| --- | --- |

Display This Question:

If If How do you know this person(s) with COVID-19? q://QID125610082/SelectedChoicesCount Is Greater Than 1

And How do you know this person(s) with COVID-19? = Myself

Or Or How do you know this person(s) with COVID-19? q://QID125610082/SelectedChoicesCount Is Greater Than 0

And How do you know this person(s) with COVID-19? != Myself

Carry Forward Selected Choices - Entered Text from "How do you know this person(s) with COVID-19?"

| 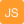 | 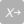 |
| --- | --- |

Q33 Did this person(s) lose his or her life as a result of COVID-19?

|  | Selection | | |
| --- | --- | --- | --- |
|  | Yes (1) | No (2) | I don't know (3) |
| Myself (x11) |  |  |  |
| My partner (x2) |  |  |  |
| My child(ren) (x3) |  |  |  |
| My parent(s) (x4) |  |  |  |
| Other family member(s) (please specify) (x5) |  |  |  |
| Friend(s) (x6) |  |  |  |
| Acquaintance(s) (x7) |  |  |  |
| Coworker(s) (x8) |  |  |  |
| Classmate(s) (x9) |  |  |  |
| Other (please specify) (x10) |  |  |  |

End of Block: Covid

Start of Block: Comorbidity

Q34 The following is a list of common health problems. Please indicate if you currently have any of the listed problems. If you have a medical condition not on the list, please list it under "other."

- Heart disease (1)
- High blood pressure (2)
- Lung disease (3)
- Diabetes (4)
- Ulcer or stomach disease (5)
- Kidney disease (6)
- Cancer (7)
- Depression (8)
- Osteoarthritis, degenerative arthritis (9)
- Back pain (10)
- Rheumatoid arthritis (11)
- Other (12) ________________________________________________

Display This Question:

If If The following is a list of common health problems. Please indicate if you currently have any of t... q://QID26/SelectedChoicesCount Is Greater Than 0

Carry Forward Selected Choices - Entered Text from "The following is a list of common health problems. Please indicate if you currently have any of the listed problems. If you have a medical condition not on the list, please list it under "other.""

| 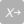 |
| --- |

Q35 Do you receive treatment for it?

|  | Selection | |
| --- | --- | --- |
|  | Yes (1) | No (2) |
| Heart disease (x1) |  |  |
| High blood pressure (x2) |  |  |
| Lung disease (x3) |  |  |
| Diabetes (x4) |  |  |
| Ulcer or stomach disease (x5) |  |  |
| Kidney disease (x6) |  |  |
| Cancer (x7) |  |  |
| Depression (x8) |  |  |
| Osteoarthritis, degenerative arthritis (x9) |  |  |
| Back pain (x10) |  |  |
| Rheumatoid arthritis (x11) |  |  |
| Other (x12) |  |  |

Display This Question:

If If The following is a list of common health problems. Please indicate if you currently have any of t... q://QID26/SelectedChoicesCount Is Greater Than 0

Carry Forward All Choices - Displayed & Hidden from "Do you receive treatment for it?"

| 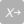 |
| --- |

Q36 Does it limit your activities?

|  | Selection | |
| --- | --- | --- |
|  | Yes (1) | No (2) |
| Heart disease (xx1) |  |  |
| High blood pressure (xx2) |  |  |
| Lung disease (xx3) |  |  |
| Diabetes (xx4) |  |  |
| Ulcer or stomach disease (xx5) |  |  |
| Kidney disease (xx6) |  |  |
| Cancer (xx7) |  |  |
| Depression (xx8) |  |  |
| Osteoarthritis, degenerative arthritis (xx9) |  |  |
| Back pain (xx10) |  |  |
| Rheumatoid arthritis (xx11) |  |  |
| Other (xx12) |  |  |

End of Block: Comorbidity

Start of Block: Additional_comments

Q37
Additional comments.

We would like to know how the pandemic has affected your life. Please tell us how you are feeling, how you are coping, or anything else you would like to share.

If you have a partner, please let us know how he or she may be helping you cope during the pandemic.

________________________________________________________________

________________________________________________________________

________________________________________________________________

________________________________________________________________

________________________________________________________________

End of Block: Additional_comments

Start of Block: Email_request

Q38
To further understand mental health during the pandemic, we would like to interview some of the participants who completed this questionnaire. If you are willing to be contacted to schedule an interview, please provide your email address. Only some participants will be contacted.
The interviews are over the phone, take approximately 30 minutes, and we will give you a gift card for your participation (20 CAD or 15 USD).
 
Please note that providing your email address here will record it with the rest of your responses to this survey (making the survey not anonymous). We will only access your email address if we plan to directly ask you to participate in an interview and will not use it for any other purpose.

- Yes, I am willing to be contacted. Email address: (1) ________________________________________________
- No, I do not want to be contacted (2)
